# The association between daily total physical activity and risk of cardiovascular disease among hypertensive patients: a 10-year prospective cohort study in China

**DOI:** 10.1186/s12889-021-10551-z

**Published:** 2021-03-16

**Authors:** Tingyu Zhou, Jian Su, Ran Tao, Yu Qin, Jinyi Zhou, Yan Lu, Yujie Hua, Jianrong Jin, Yu Guo, Zhengming Chen, Liming Li, Ming Wu

**Affiliations:** 1 Department of Epidemiology and Biostatistics, School of Public Health, Nanjing Medical University, Nanjing, 211166 China; 2 Department of Non-communicable Chronic Disease Control and Prevention, Jiangsu Provincial Center for Disease Control and Prevention, Nanjing, 210009 China; 3 Department of Non-communicable Chronic Disease Control and Prevention, Suzhou City Center for Disease Control and Prevention, Suzhou, 215004 China; 4 Wuzhong District of Suzhou City Center for Disease Control and Prevention, Suzhou, 215100 China; 5 Chinese Academy of Medical Sciences, Beijing, 102308 China; 6 Clinical Trial Service Unit & Epidemiological Studies Unit (CTSU), Nuffield Department of Population Health, University of Oxford, Oxford, OX3 7LF UK; 7 Department of Epidemiology and Biostatistics, School of Public Health, Peking University, Beijing, 100191 China

**Keywords:** China, Physical activity, Hypertension, Cardiovascular disease

## Abstract

**Background:**

The effect of high levels of physical activity and relationship between daily total physical activity and the risk of cardiovascular disease (CVD) among hypertensive people were not clear. This study aimed to explore the optimum level of physical activity for CVD prevention.

**Methods:**

Data used in the present study was derived from the sub-study of China Kadoorie Biobank study (CKB) in Jiangsu province of China. The CKB was a prospective cohort study established during 2004–2008. At baseline, 53,259 participants aged 35–74 years were recruited for the CKB Jiangsu sub-study conducted in Wuzhong district of Suzhou City. Among those 53,259 participants, the 20,179 hypertensive individuals were our study population. The outcome events were cardiovascular diseases (CVDs), while the independent variable was total daily physical activity. The Cox proportional hazard models were introduced to investigate the association between total physical activity and CVDs, reporting as hazard ratios (HR) and 95% confidence intervals (CIs).

**Results:**

During a 10.1-year follow-up, 2419 CVD cases were identified. After adjustment for potential confounding factors, compared with participants at the lowest level of daily total physical activity, the hazard ratios for CVDs were 0.87 (95%CI: 0.79–0.97), 0.73 (95%CI: 0.65–0.83) and 0.75 (95%CI: 0.65–0.85) for participants within 2, 3 and 4 quartiles of physical activity. Such a negative association between total physical activity and CVDs were also observed among participants by gender and age-group, but within patients with stage 1 hypertension only. Moreover, the association of physical activity with CVDs was U-shape and the lowest HR (0.63, 95%CI: 0.54–0.74) was observed at 35.4 MET-h/d of total physical activity.

**Conclusions:**

Total daily physical activity was negatively associated with CVDs among hypertensive adults in China, and this association was U-shape. It has some public health implications that community-based total physical activity intervention campaigns can be of help for CVDs prevention among hypertensive people in China.

**Supplementary Information:**

The online version contains supplementary material available at 10.1186/s12889-021-10551-z.

## Introduction

The number of adults with hypertension has tripled during the past 40 years in low- and middle-income countries [1]. In China, a large nationwide representative survey reported that 23.2% of Chinese adults had hypertension in 2012–2015 [2], while uncontrolled blood pressure (BP) among hypertensive patients was found to contribute to approximately one-third death of cardiovascular diseases (CVDs) [3]. Hypertensive patients are at a higher risk of experiencing CVD events and appear to experience CVD events about 5 years earlier than their counterparts with blood pressure under control [4].

The hypertension-induced CVD death may be prevented through improving physical activity (PA), as evidence show that PA can reduce the risk of hypertension [5, 6]. PA can provide a wide range of favorable response in cardiovascular system such as improving the dynamics of cardiovascular system and reducing the prevalence of coronary heart disease and cardiomyopathy [7]. One study conducted by O’Donovan et al. reported that compared with physically inactive participants, the hazard ratio (HR) for CVD mortality was 0.59 (95% CI: 0.48–0.73) for regularly active participants [8]. Despite the repeated evidence on the inverse association between PA and mortality risk of CVD in general population [9], such an association among hypertensive patients remains scarce.

The 2018 European Society of Cardiology/Hypertension (ESC/ESH) guidelines for the arterial hypertension recommended regular aerobic exercise, at least 30-min of moderate dynamic aerobic exercise for 5–7 days per week, i.e., at least 150 min/week [10], while physical activity was generally not recommended for hypertensive persons with very high BP (i.e., BP > 180/110 mmHg) [11]. At meantime, hypertensive people appear to be less physically active than those without hypertension [12]. It is reported that only about one third of hypertensive patients achieve the recommended PA level [13]. Most previous researches subdivided physical activity by domains (e.g., occupational, domestic and leisure-time activity) [14] or intensities (e.g., light, moderate and vigorous physical activities) when exploring the relationship between PA and CVDs [15]. However, in developing countries including China, residents engaged in physical activity with occupational and domestic activity as the majority but very few PA at leisure time [16]. Therefore, daily total physical activity might better reflect the level of physical activity compared with selected domains or intensities of physical activity for people in developing societies. To bridge this gap, we used data from a 10-year prospective cohort study in China to examine the relationship between daily total physical activity and reduced risk of cardiovascular diseases among hypertensive individuals in China.

## Methods

### Study design and participants

The data used in this study were derived from the China Kadoorie Biobank (CKB) study in Wuzhong district of Suzhou city, Jiangsu Province. Details of the study design, survey methods, and participant characteristics have been reported elsewhere previously [17, 18]. CKB study was conducted in 10 geographically defined regions (5 urban and 5 rural) in China, and Wuzhong district of Suzhou is one of the five urban regions. Between June 2004 and July 2008, 53,259 of permanent residents aged 35–74 years with no severe disability were recruited for the baseline survey. Subsequently, participants were followed up for morbidity and mortality every year. By January 14 of 2017, there were 2934 death and 476 (< 1%) participants lost to follow-up.

In the present study, we included subjects with prevalent hypertension at baseline. Prevalent hypertensive individuals were defined as those: (a) with measured systolic BP (SBP) ≥140 mmHg, and/or measured diastolic BP (DBP) ≥90 mmHg, or (b) having been previously diagnosed as hypertensive patients by registered physicians, or (c) using antihypertensives [3]. Initially, 21,124 participants with hypertension were successfully recruited. Among them, 945 individuals were not included in our analysis as they had been diagnosed with CVDs, e.g., stroke, heart disease, or cancer at baseline and thus 20,179 hypertensive participants were included in the present study.

### Ascertainment of outcomes

The information on incidence of CVD was regularly collected through Disease Surveillance Points (DSP) system death registry, chronic disease registry, national health insurance system, health insurance databases and annual active follow-up [17]. Each CVD event was ascertained by scanned copies of medical records, original disease reporting card or official death certificates [19]. There was a city-wide investigation on under-reporting rate of death cases in disease surveillance system every 3 years in Suzhou to assess the under-reporting of death cases, and the under-reporting rates were < 5% in Suzhou in the past 10 years [20]. Fatal and nonfatal events were coded using the International Classification of Disease, Tenth Revision (ICD-10) by trained staff “blinded” to baseline exposures. The primary outcome events were occurring ischemic heart disease (IHD) (I20–25) and cerebrovascular diseases (I60-I69). In the analysis, participants were classified as having or not having CVDs during the follow-up period.

### Physical activity measurements

At baseline survey, the information on physical activity included frequency, intensity, and time spent on occupational tasks, commuting, leisure time activities and household tasks during the past year. Occupational tasks refer to the physical activity performed during paid employment, while commuting PA means the physical activity performed during travel to and from daily work. The time spent on leisure-sedentary activities such as watching television, playing cards and reading was defined as leisure-sedentary time. The questions used to measure physical activity were adapted from validated instruments used in previous studies [21, 22]. Those adapted PA question items were then integrated into a questionnaire specifically for assessing physical activity in CKB study [23], although it was not validated as a survey instrument.

For all the four PA domains, a specific metabolic equivalent (MET) was assigned to each PA type. Metabolic equivalents of each PA type were adopted from the 2011 Compendium of Physical Activities [24]. The sum of all the METs/day was defined as the total daily PA in this study. And then participants were quartiled into four sub-groups for analysis: Q1 (< 11.20 MET-h/d), Q2 (11.20–19.99 MET-h/d), Q3 (20.00–34.71 MET-h/d) and Q4 (> 34.71 MET-h/d).

### BP measurements

All participants were asked to remain at rest in a seated position for at least 5 min before BP measurement. Additionally, smokers were required not to smoke for at least 15 min. BP was measured twice using a UA-779 digital monitor on unclothed right upper arm. If the variation of SBP between two measurements differed by > 10 mmHg, a third measurement would be taken and the last two measurements were recorded. We used the mean of the two BP readings in the analysis.

The BP-levels were classified according to Chinese Guidelines for Prevention and Treatment of Hypertension using records of on-site measurement at baseline survey [25]. For those hypertensive individuals, they were classified as having BP under control, if they had SBP < 140 mmHg and DBP < 90 mmHg. Definitions of other BP-levels for hypertensive participants are presented below:

1. Stage 1 hypertension: SBP between 140 and 159 mmHg or DBP between 90 and 99 mmHg.
2. Stage 2 hypertension: SBP between 160 and 179 mmHg or DBP between 100 and 109 mmHg.
3. Stage 3 hypertension: SBP ≥180 mmHg or DBP ≥110 mmHg.

### Covariates measurements

The survey was conducted via face-to-face interview by trained health workers using a laptop-based questionnaire. Information on covariates included sociodemographic characteristics (age, gender, levels of education etc), lifestyle behaviors (smoking status, alcohol consumption, intakes of red meat and fresh fruit etc), personal health and medical history (hypertension and diabetes).

Smoking status and alcohol consumption were classified as current smoker/weekly drinker or not in this study. Current smokers were defined as those who reported having ever smoked one or more cigarettes (or their equivalent) daily for at least 6 months [26]. Current drinkers were specified as those who had drunk alcohol at least once a week during the previous 12 months [27]. Measurements of weight, height, waist and hip circumferences were conducted at interview. Body mass index (BMI) was calculated as BMI = (body weight in kilograms)/(height (in meters)^2^). Diabetes mellitus was defined as a self-reported of physician diagnosis or screen-detected diabetes [28]. Screen-detected diabetes was defined as no prior history of diabetes with either non-fasting blood glucose ≥11.1 mmol/L or fasting blood glucose ≥7.0 mmol/L [29].

### Statistical analysis

Summary statistics of demographics were reported as mean ± standard deviation for numerical variables. Continuous variables were compared by one-way analysis of variance and using Welch’s ANOVA when variances were unequal. Categorical variables were compared by Pearson’s χ^2^ test between different levels of physical activity groups. Person-years at risk were calculated from the recruitment date at baseline to the date of death, loss to follow-up, or censoring date, whichever came first. The Cox proportional hazard models were used to identify the association between levels of total physical activity and incidence of CVD [30] by hazard ratio (HR) and 95% confidence interval (CI). Covariates adjusted for in multivariable models included age at baseline (4 groups), gender (male or female), smoking status (current smoker or not), alcohol consumption (current weekly drinker or not), intake frequencies of red meat and fresh fruit (< 4 times per week or ≥ 4 times per week), prevalent diabetes at baseline (presence or absence), leisure-sedentary time (continuous) and BP-levels (4 groups) [31]. Sensitivity analyses excluded participants who had IHD and cerebrovascular diseases during the first year were conducted in the study.

Linear relationship between daily total physical activity and CVD risk was assessed using restricted cubic spline functions [15] with three knots located at the 5th, 50th, and 95th percentiles of physical activity. SAS 9.4 (SAS Institute, Cary, NC) and STATA 13.0 (Stata Corp, College Station, TX, USA) were used for the statistical analyses. All statistical tests were 2-tailed and *P* value less than 0.05 was regarded as significant.

## Results

### Baseline characteristics of participants

Among the 20,179 participants at baseline, their mean age was 56.3 ± 9.8 years, and 44.3% were men. And, those who reported higher levels of total physical activity tended to be younger, more likely to be male, with less diabetes and lower SBP level (Table 1). There were very few people (0.8%) people lost to follow-up. During 211,066 person-years of follow-up, there were 2419 patients of CVD observed in this study, including 1672 people with cerebrovascular disease, 584 individuals with IHD, and 163 participants simultaneously with cerebrovascular disease and IHD.

**Table 1:** Selected characteristics of participants by the quartiles of physical activity in this study

Characteristics		Physical activity				
	*N* = 20,179	Q1 *n* = 4919	Q2 *n* = 5166	Q3 *n* = 5046	Q4 *n* = 5048	*P* value
Age. y	56.3 ± 9.8	62.2 ± 8.9	57.4 ± 9.4	53.8 ± 9.1	51.7 ± 8.4	< 0.001^*^
Male, No. (%)	8941 (44.3)	2106 (42.8)	1733 (33.6)	2145 (42.5)	2957 (58.6)	< 0.001^*^
Treated with antihypertension, No. (%)	8134 (40.3)	2460 (50.0)	2264 (43.8)	1820 (36.1)	1590 (31.5)	< 0.001^*^
Diabetes mellitus, No. (%)	1690 (8.4)	562 (11.4)	489 (9.5)	359 (7.1)	280 (5.6)	< 0.001^*^
SBP, mmHg	150.9 ± 18.1	151.6 ± 19.1	151.2 ± 18.6	151.1 ± 18.1	149.5 ± 16.6	< 0.001^*^
DBP, mmHg	86.3 ± 9.9	84.4 ± 10.3	85.6 ± 9.9	87.3 ± 9.7	87.9 ± 9.5	< 0.001^*^
BMI, Kg/m^2^	25.0 ± 3.3	24.8 ± 3.4	25.2 ± 3.3	25.1 ± 3.3	25.0 ± 3.1	0.088
Resting heart rate, bpm	80.8 ± 13.1	80.6 ± 13.1	80.9 ± 13.1	80.9 ± 13.3	80.7 ± 13.0	0.385
Leisure-sedentary time, h/d	3.1 ± 2.2	3.8 ± 2.9	3.3 ± 2.2	2.7 ± 1.6	2.4 ± 1.4	< 0.001^*^
Current smoker, No. (%)	5745 (28.5)	1151 (23.4)	1109 (21.5)	1447 (28.7)	2038 (40.4)	< 0.001^*^
Current weekly drinker, No. (%)	3938 (19.5)	733 (14.9)	766 (14.8)	979 (19.4)	1460 (28.9)	< 0.001^*^
Education, No. (%)						< 0.001^*^
≤ 6y	14,149 (70.1)	3758 (76.4)	3536 (68.5)	3472 (68.8)	3383 (67.0)	
7-9y	4270 (21.2)	710 (14.4)	1018 (19.7)	1168 (23.2)	1374 (27.2)	
10-12y	1364 (6.7)	316 (6.5)	446 (8.6)	334 (6.6)	268 (5.3)	
≥ 13y	396 (2.0)	135 (2.7)	166 (3.2)	72 (1.4)	23 (0.5)	

(1) Mean and standard deviation, unless specified otherwise

(2) * *P* < 0.05

In general, levels of total physical activity (mean [SD]) among men were higher than women (25.7 [16.4] vs 21.2 [13.8] MET-h/d, *P* < 0.01), while men had higher occupational physical activity (19.9 [16.3] vs 11.2 [14.0] MET-h/d, *P* < 0.01), but less household activity (3.4 [3.1] vs 8.5 [5.2] MET-h/d, *P* < 0.01) than women. The level of physical activity engaged in leisure-time activity among men was higher than that among women (0.8 [2.1] vs 0.5 [1.7] MET-h/d, *P* < 0.01).

### Association of total physical activity with risk of CVD

Table 2 presents the association of total daily PA and the risk of developing CVDs among overall and gender-stratified participants in this study. Total physical activity levels were inversely associated with the risk of CVDs. Compared with hypertensive patients in the lowest quartile of physical activity, the hazard ratios (95%CIs) after adjustment for potential confounding factors were 0.87 (0.79–0.97), 0.73 (0.65–0.83) and 0.75 (0.65–0.85) for those in quartile 2, 3 and 4, respectively. After stratified by gender, the significant relationship between PA and CVDs were still examined among men and women, with one exception that the association between Q2 PA and CVDs among women was not significant. Moreover, the similar scenarios were also observed for participants within different age-groups (Table 3).

**Table 2:** Hazard ratios (95% CI) of CVD across quartiles of total physical activity for all participants and stratified by gender in this study

Physical activity	No. of Observations	No. of Cases	Person years of follow-up	Incidence density (1/1000 person-years)	Model 1 Hazard ratio (95%CI)	*P* value	Model 2 Hazard ratio (95%CI)	*P* value
All	20,179	2419	211,066	11.46				
Q1	4919	943	50,392	18.71	1.00		1.00	
Q2	5166	651	54,255	12.00	0.86 (0.78–0.96)	0.005*	0.87 (0.79–0.97)	0.009*
Q3	5046	439	53,535	8.20	0.72 (0.64–0.81)	< 0.001*	0.73 (0.65–0.83)	< 0.001*
Q4	5048	386	52,884	7.30	0.72 (0.63–0.82)	< 0.001*	0.75 (0.65–0.85)	< 0.001*
Male	8941	1135	91,136	12.45				
Q1	2106	452	20,758	21.77	1.00		1.00	
Q2	1733	218	17,780	12.26	0.82 (0.69–0.96)	0.016*	0.82 (0.69–0.97)	0.017*
Q3	2145	200	22,133	9.04	0.70 (0.59–0.84)	< 0.001*	0.70 (0.58–0.83)	< 0.001*
Q4	2957	265	30,465	8.70	0.72 (0.61–0.85)	< 0.001*	0.73 (0.61–0.87)	< 0.001*
Female	11,238	1284	119,930	10.71				
Q1	2813	491	29,634	16.57	1.00		1.00	
Q2	3433	433	36,475	11.87	0.89 (0.78–1.02)	0.086	0.91 (0.80–1.04)	0.153
Q3	2901	239	31,402	7.61	0.73 (0.63–0.86)	< 0.001*	0.76 (0.65–0.90)	0.001*
Q4	2091	121	22,419	5.40	0.70 (0.57–0.86)	< 0.001*	0.75 (0.60–0.93)	0.008*

Model 1 was adjusted for age and gender

Model 2 was additionally adjusted for smoking status, alcohol consumption, intake frequencies of red meat, intake frequencies of fresh fruit, prevalent diabetes at baseline, leisure-sedentary time and BP-levels

* *P* < 0.05

**Table 3:** Hazard ratios (95% CI) of CVD across quartiles of total physical activity stratified by age in this study

	No. of Observations	No. of Cases	Person years of follow-up	Incidence density (1/1000 person-years)	Model 1 Hazard ratio (95%CI)	*P* value	Model 2 Hazard ratio (95%CI)	*P* value
Age (years)								
< 50	5038	181	53,207	3.40				
Q1	464	30	4976	6.03	1.00		1.00	
Q2	1005	34	10,574	3.22	0.53 (0.33–0.87)	0.012*	0.58 (0.35–0.97)	0.037*
Q3	1580	46	16,701	2.75	0.46 (0.29–0.72)	< 0.001*	0.51 (0.31–0.82)	0.006*
Q4	1989	71	20,956	3.39	0.56 (0.37–0.86)	0.008*	0.63 (0.40–1.00)	0.051
50-	7365	579	78,045	7.42				
Q1	1188	114	12,693	8.98	1.00		1.00	
Q2	1971	164	21,016	7.80	0.87 (0.69–1.11)	0.260	0.91 (0.72–1.16)	0.440
Q3	2084	140	22,157	6.32	0.67 (0.52–0.86)	0.001*	0.70 (0.54–0.91)	0.007*
Q4	2122	161	22,179	7.26	0.72 (0.56–0.92)	0.008*	0.79 (0.61–1.02)	0.071
60-	5666	1027	59,177	17.35				
Q1	2081	429	21,372	20.07	1.00		1.00	
Q2	1600	272	16,770	16.22	0.82 (0.70–0.96)	0.012*	0.83 (0.71–0.96)	0.015*
Q3	1143	196	12,274	15.97	0.78 (0.66–0.92)	0.004*	0.78 (0.66–0.93)	0.006*
Q4	842	130	8761	14.84	0.67 (0.55–0.82)	< 0.001*	0.69 (0.56–0.85)	< 0.001*
≥ 70	2110	632	20,637	30.62				
Q1	1186	370	11,351	32.60	1.00		1.00	
Q2	590	181	5895	30.70	0.96 (0.80–1.15)	0.674	0.97 (0.81–1.16)	0.712
Q3	239	57	2403	23.72	0.71 (0.54–0.94)	0.016*	0.70 (0.53–0.93)	0.014*
Q4	95	24	988	24.29	0.70 (0.47–1.07)	0.097	0.71 (0.47–1.09)	0.115

Model 1 was adjusted for gender

Model 2 was additionally adjusted for smoking status, alcohol consumption, intake frequencies of red meat, intake frequencies of fresh fruit, prevalent diabetes at baseline, leisure-sedentary time and BP-levels

* *P* < 0.05

Table 4 shows the associations of PA with CVDs among participants with different BP levels. Interestingly, the significant associations of PA with CVDs were identified only among stage 1 hypertensive patients, showing a reduction of CVD risk of 22%, 41% and 34% for participants within quartile 2, 3 and 4, respectively, compared to those with BP under-control. Moreover, participants with stage 3 hypertension within Q3 PA sub-group were less likely to develop CVDs (HR = 0.67, 95%CI: 0.49–0.96) relative to their counterparts within Q1 category. We also took a look at the PA-CVDs associations within participants who had self-reported hypertension and newly-detected hypertension, separately. The scenarios were similar to those examined among overall participants (Additional file 1: Appendix 1).

**Table 4:** Hazard ratios (95%CI) of CVD across quartiles of total physical activity stratified by BP-levels in this study

	No. of Observations	No. of Cases	Person years of follow-up	Incidence density (1/1000 person-years)	Model 1 Hazard ratio (95%CI)	*P* value	Model 2 Hazard ratio (95%CI)	*P* value
BP under control	3141	406	32,394	12.53				
Q1	912	167	9235	18.08	1.00		1.00	
Q2	853	116	8873	13.07	1.01 (0.79–1.29)	0.927	1.04 (0.81–1.33)	0.759
Q3	690	66	7217	9.15	0.86 (0.64–1.16)	0.321	0.88 (0.65–1.19)	0.410
Q4	686	57	7069	8.06	0.80 (0.58–1.10)	0.170	0.82 (0.59–1.15)	0.253
Stage 1 hypertension	11,508	1106	121,101	9.13				
Q1	2536	437	26,195	16.68	1.00		1.00	
Q2	2857	290	30,126	9.63	0.77 (0.67–0.90)	< 0.001*	0.78 (0.67–0.91)	0.001*
Q3	2969	185	31,634	5.85	0.58 (0.48–0.69)	< 0.001*	0.59 (0.49–0.71)	< 0.001*
Q4	3146	194	33,146	5.85	0.64 (0.53–0.77)	< 0.001*	0.66 (0.55–0.80)	< 0.001*
Stage 2 hypertension	4048	594	42,272	14.05				
Q1	1057	214	10,833	19.75	1.00		1.00	
Q2	1051	164	11,009	14.90	0.99 (0.81–1.22)	0.938	1.00 (0.81–1.23)	0.974
Q3	1015	131	10,777	12.16	0.98 (0.78–1.23)	0.864	1.01 (0.80–1.27)	0.957
Q4	925	85	9653	8.81	0.79 (0.60–1.04)	0.095	0.83 (0.62–1.10)	0.192
Stage 3 hypertension	1482	313	15,299	20.46				
Q1	414	125	4129	30.27	1.00		1.00	
Q2	405	81	4247	19.07	0.79 (0.59–1.05)	0.106	0.80 (0.60–1.07)	0.129
Q3	372	57	3907	14.59	0.65 (0.47–0.91)	0.011*	0.67 (0.49–0.96)	0.027*
Q4	291	50	3016	16.58	0.87 (0.60–1.25)	0.442	0.89 (0.61–1.30)	0.552

Model 1 was adjusted for age and gender

Model 2 was additionally adjusted for smoking status, alcohol consumption, intake frequencies of red meat, intake frequencies of fresh fruit, prevalent diabetes at baseline and leisure-sedentary time

* *P* < 0.05

Further, we classified individuals into three categories according to the latest WHO guidelines on physical activity: insufficient physical activity (group 1), meet the weekly physical activity recommendation (group 2) and exceed the recommendations of the guidelines (group 3) [32]. Only those participants who met the physical activity recommendation had the lowest risk of CVD (HR = 0.80, 95%CI: 0.69–0.92) (Additional file 1: Appendix 2). The associations of total physical activity and risk of CVD did not change substantially in sensitivity analyses with exclusion of participants who had CVD during the first year of follow-up (Additional file 1: Appendix 3–5).

**Fig. 1:**
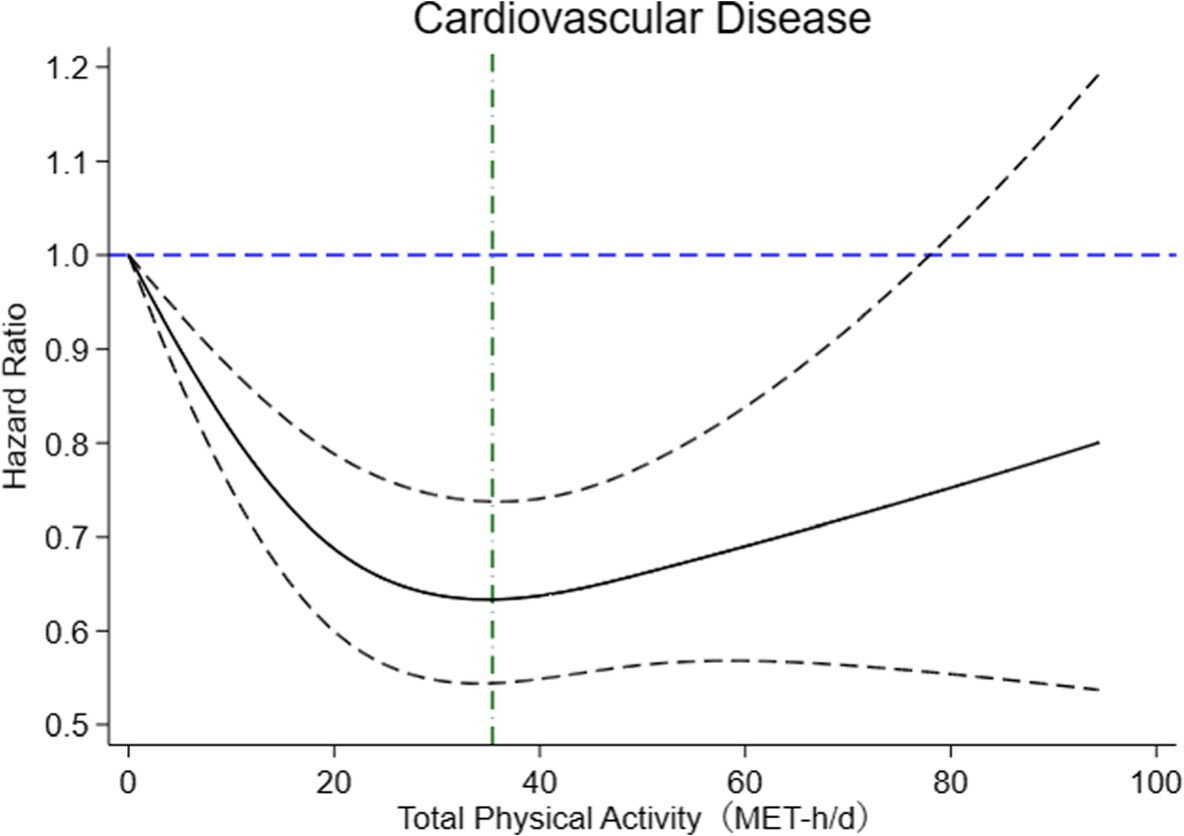
Restricted cubic spline model of hazard ration of CVD with total physical activity. Adjust for gender, age, smoking status, alcohol consumption, intake frequencies of red meat, intake frequencies of fresh fruit, prevalent diabetes at baseline, leisure-sedentary time and BP-levels. The dashed lines represent the 95% confidence intervals

Figure 1 displays the linear relations between daily total physical activity level and CVDs. Cubic spline graphs revealed a U-shape relationship between PA and CVDs among participants, although PA was negatively associated with CVDs in this study. When participants had a total daily PA of 35.4 MET-h/d, they were at the lowest risk of experiencing CVDs (HR = 0.63, 95%CI: 0.54–0.74).

## Discussion

In this prospective population-based study among Chinese hypertensive adults, we aimed to examine the association of total daily PA and the risk of developing CVDs. We found that being physically active was significantly associated with a reduced risk of CVDs, and such a significant association between PA and CVDs was mainly among patients with stage 1 hypertension. Moreover, the association between total physical activity level and CVD risk was U-shape, suggesting that an optimal PA level existed for prevention of CVDs among hypertensive adults.

To date, several studies have evaluated the beneficial impact of sufficient physical activity on hypertensive individuals [33, 34]. Fan et al. reported that high level of physical activity group had a 35% risk reduction of death from CVD among Chinese population [31]. And one study conducted among 18,794 Danish hypertensive people showed that moderate/high level of physical activity might prevent participants from CVD-induced death with a dose-response relationship [11]. The findings in our study were in line with those documented in the previous literature [11, 31, 33, 34]. The interesting finding that a U-shape relationship between PA and CVDs was also similar to that reported in a study conducted among Japanese population [35]. In this Japanese study, a U- or J-shape association of PA with hemorrhage stroke and a L-shape association with ischemic stroke were identified [35]. This might be, at least partially, explained by that high-intensity physical activity can cause a short-lasting and sudden increase in BP [36] and lead to temporary physiological pressure [37].

A recent meta-analysis showed that 4000–7999 MET-minutes per week, equivalent to 10–19 MET-h/d, could substantially reduce the risk of IHD (23%) [38]. Another meta-analysis included 36 studies reported that the greatest reduction of CVD risk was observed from inactive to moderate physical activity [39]. In our study, the optimal effect of PA on CVDs was observed at 35.4 MET-h/d, however participants could get the major benefit of total physical activity on CVDs with 7–25 MET-h/d.

Physical activity was documented as one of the influencing factors of and cornerstone therapies for reducing risk of CVD in patients with hypertension [40]. The mechanisms might involve the exercise-induced cardiovascular benefits, which prevent age-related decrements in arterial compliance and function [41]. Regular exercise might be able to reduce circulate markers of systemic inflammation [42], improve calcium handling through the sarcoendoplasmic reticulum calcium transport ATPase [43] and reduce the production of reactive oxygen species in mitochondrial [44].

### Strengths and limitations

The present study has several strengths. First, the data used in this study was from a 10-yearfollow-up prospective cohort study, suggesting the association between physical activity and CVDs was of a causal nature. Second, total physical activity, not selected PA domains, was used to predict participants’ PA level. Third, the classical potential confounding factors of CVDs were adjusted for in the analysis.

Meanwhile, there are some limitations deserved attention for the interpretation of our study finding. First, only physician-diagnosed CVDs were defined as the outcome events. If CVD patients had mild symptoms but were not recorded in hospitalization registry, they might be not classified as outcome case. This may lead to an underestimation of the PA-CVDs association. Second, the question items used to assess physical activity were adapted from validated instruments and integrated into a PA questionnaire, however this integrated PA questionnaire was not specifically validated for CKB study. Third, information on physical activity was self-reported, which may imply potential recall bias. Fourth, the baseline information on physical activity was used as independent variable. Over the 10-yearfollow-up period, participants PA level might different from that at baseline. Therefore, the findings from our study should be interpreted prudently.

## Conclusions

In conclusion, there is a significant negative association between total daily physical activity and CVDs and such a negative association was U-shape among hypertensive patients in China. It has some public health implications that community-based total physical activity intervention campaigns can be of help for CVDs prevention among hypertensive people in China.

## Supplementary Information

**Additional file 1 **: **Appendix 1**. Hazard ratios (95%CI) of CVD across quartiles of total physical activity stratified by BP-levels with self-reported and new detected hypertension. **Appendix 2**. Hazard ratios (95% CI) of CVD across physical activity categorized by recommendation of guidelines. **Appendix 3**. Hazard ratios (95% CI) of CVD across quartiles of total physical activity for all participants and stratified by gender with sensitivity analyses. **Appendix 4**. Hazard ratios (95%CI) of CVD across quartiles of total physical activity stratified by age with sensitivity analyses. **Appendix 5.** Hazard ratios (95%CI) of CVD across quartiles of total physical activity stratified by BP-levels with sensitivity analyses. **Appendix 6**. Basic characteristics of respondent, non-respondent, death population of Respondent and respondents die of CVD at baseline.

## Data Availability

The data that support the findings of this study are available from Department of China Kadoorie Biobank but restrictions apply to the availability of these data, which were used under license for the current study, and so are not publicly available. Data are however available from the authors upon reasonable request and with permission of Department of China Kadoorie Biobank.
