# Supplementary material for: The association between daily total physical activity and risk of cardiovascular disease among hypertensive patients: a 10-year prospective cohort study in China
Source: BMC Public Health. 2021 Mar 16;21:517. doi: 10.1186/s12889-021-10551-z (PMC7968198; doi:10.1186/s12889-021-10551-z)
Supplement: Supplementary file 1 — Additional file 1 : Appendix 1. Hazard ratios (95%CI) of CVD across quartiles of total physical activity stratified by BP-levels with self-reported and new detected hypertension. Appendix 2. Hazard ratios (95% CI) of CVD across physical activity categorized by recommendation of guidelines. Appendix 3. Hazard ratios (95% CI) of CVD across quartiles of total physical activity for all participants and stratified by gender with sensitivity analyses. Appendix 4. Hazard ratios (95%CI) of CVD across quartiles of total physical activity stratified by age with sensitivity analyses. Appendix 5. Hazard ratios (95%CI) of CVD across quartiles of total physical activity stratified by BP-levels with sensitivity analyses. Appendix 6. Basic characteristics of respondent, non-respondent, death population of Respondent and respondents die of CVD at baseline. [file 12889_2021_10551_MOESM1_ESM.docx]

**The association between daily total physical activity and risk of cardiovascular disease among hypertensive patients: a 10-year prospective cohort study in China**

Tingyu Zhou^a^, Jian Su^b^, Ran Tao^b^, Yu Qin^b^, Jinyi Zhou^b^, Yan Lu^c^, Yujie Hua^c^, Jianrong Jin^d^, Yu Guo^e^, Zhengming Chen^f^, Liming Li^g^, Ming Wu^a,b^

^a^Department of Epidemiology and Biostatistics, School of Public Health, Nanjing Medical University, Nanjing 211166, China

^b^Department of Non-communicable Chronic Disease Control, Jiangsu Provincial Center for Disease Control and Prevention, Nanjing 210009, China

^c^Department of Non-communicable Chronic Disease Control, Suzhou Center for Disease Control and Prevention, Suzhou 215004, China

^d^Wuzhong Center for Disease Control and Prevention, Suzhou 215100, China

^e^Chinese Academy of Medical Sciences, Beijing 102308, China

^f^Clinical Trial Service Unit & Epidemiological Studies Unit (CTSU), Nuffield Department of Population Health, University of Oxford, Oxford OX3 7LF, United Kingdom

^g^Department of Epidemiology and Biostatistics, School of Public Health, Peking University Health Science Center, Beijing 100191, China

Correspondence should be addressed to Ming Wu; jswuming@vip.sina.com

Tingyu Zhou and Jian Su contributed equally to this work.

**Appendix 1. Hazard ratios (95%CI) of CVD across quartiles of total physical activity stratified by BP-levels with** **self-reported and new detected hypertension**

|  | No. of Observations | No. of  Cases | Person years of follow-up | Incidence density (1/1000 person-years) | Model 1  Hazard ratio (95%CI) | *P* value | Model 2  Hazard ratio (95%CI) | *P* value | |
| --- | --- | --- | --- | --- | --- | --- | --- | --- | --- |
| **Self-reported hypertension group** | | | | | | | | |  |
| BP under control | 3141 | 406 | 32394 | 12.53 |  |  |  |  |  |
| Q1 | 912 | 167 | 9235 | 18.08 | 1.00 |  | 1.00 |  |  |
| Q2 | 853 | 116 | 8873 | 13.07 | 1.01 (0.79-1.29) | 0.927 | 1.04 (0.81-1.33) | 0.759 |  |
| Q3  Q4 | 690  686 | 66  57 | 7217  7069 | 9.15  8.06 | 0.86 (0.64-1.16)  0.80 (0.58-1.10) | 0.321  0.170 | 0.88 (0.65-1.19)  0.82 (0.59-1.15) | 0.410  0.253 |  |
| Stage 1 hypertension | 3525 | 526 | 36775 | 14.30 |  |  |  |  |  |
| Q1 | 1029 | 234 | 10594 | 22.09 | 1.00 |  | 1.00 |  |  |
| Q2 | 1001 | 149 | 10462 | 14.24 | 0.79 (0.64-0.98) | 0.031* | 0.80 (0.65-0.99) | 0.036* |  |
| Q3  Q4 | 788  707 | 76  67 | 8357  7362 | 9.09  9.10 | 0.56 (0.43-0.73)  0.57 (0.43-0.76) | <0.001*  <0.001* | 0.57 (0.44-0.75)  0.60 (0.45-0.81) | <0.001*  <0.001* |  |
| Stage 2 and 3 hypertensions | 2634 | 517 | 27278 | 18.95 |  |  |  |  |  |
| Q1 | 815 | 201 | 8260 | 24.33 | 1.00 |  | 1.00 |  |  |
| Q2 | 724 | 147 | 7552 | 19.47 | 0.99 (0.80-1.24) | 0.960 | 1.01 (0.81-1.25) | 0.934 |  |
| Q3  Q4 | 615  480 | 96  73 | 6469  4997 | 14.84  14.61 | 0.86 (0.67-1.11)  0.97 (0.73-1.29) | 0.258  0.831 | 0.88 (0.68-1.14)  0.97 (0.72-1.30) | 0.321  0.817 |  |
| **Newly-detected hypertension group** | | | | | | | | |  |
| Stage 1 hypertension | 7983 | 580 | 84326 | 6.88 |  |  |  |  |  |
| Q1 | 1507 | 203 | 15601 | 13.01 | 1.00 |  | 1.00 |  |  |
| Q2 | 1856 | 141 | 19664 | 7.17 | 0.77 (0.62-0.96) | 0.018* | 0.77 (0.62-0.95) | 0.016* |  |
| Q3 | 2181 | 109 | 23277 | 4.68 | 0.65 (0.51-0.83) | <0.001* | 0.64 (0.50-0.81) | <0.001* |  |
| Q4 | 2439 | 127 | 25784 | 4.93 | 0.78 (0.62-0.99) | 0.048* | 0.76 (0.59-0.98) | 0.031* |  |
| Stage 2 and 3 hypertensions | 2896 | 390 | 30293 | 12.87 |  |  |  |  |  |
| Q1 | 656 | 138 | 6702 | 20.59 | 1.00 |  | 1.00 |  |  |
| Q2 | 732 | 98 | 7704 | 12.72 | 0.85 (0.65-1.11) | 0.232 | 0.85 (0.65-1.11) | 0.222 |  |
| Q3 | 772 | 92 | 8215 | 11.20 | 0.89 (0.67-1.18) | 0.416 | 0.93 (0.70-1.24) | 0.622 |  |
| Q4 | 736 | 62 | 7672 | 8.08 | 0.71 (0.51-0.99) | 0.044* | 0.78 (0.55-1.10) | 0.149 |  |

* *P* <0.05.

Model 1 was adjusted for age and gender.

Model 2 was additionally adjusted for smoking status, alcohol consumption, intake frequencies of red meat, intake frequencies of fresh fruit, prevalent diabetes at baseline and leisure-sedentary time.

According to the latest WHO guidelines on physical activity, it recommended adults with chronic condition should do at least 150-300 minutes of moderate-intensity aerobic physical activity; or 75-150 minutes of vigorous-intensity aerobic physical activity or an equivalent combination of moderate and vigorous-intensity activity throughout the week. We categorized individuals into three levels: insufficient physical activity (group 1), meet the weekly physical activity recommendation (group 2) and exceed the recommendations of the guidelines (group 3).

**Appendix 2. Hazard ratios (95% CI) of CVD across physical activity categorized by recommendation of guidelines**

| Physical activity | No. of Observations | No. of  Cases | Person years of follow-up | Incidence density (1/1000 person-years) | Model 1  Hazard ratio (95%CI) | *P* value | Model 2  Hazard ratio (95%CI) | *P* value |
| --- | --- | --- | --- | --- | --- | --- | --- | --- |
|  | 20179 | 2419 | 211066 | 11.46 |  |  |  |  |
| Group 1 | 11403 | 1504 | 119957 | 12.54 | 1.00 |  | 1.00 |  |
| Group 2 | 2679 | 213 | 28182 | 7.56 | 0.79 (0.69-0.92) | 0.002* | 0.80 (0.69-0.92) | 0.002* |
| Group 3 | 6097 | 702 | 62927 | 11.16 | 0.86 (0.79-0.95) | 0.002* | 0.87 (0.79-0.96) | 0.004* |

Model 1 was adjusted for age and gender.

Model 2 was additionally adjusted for smoking status, alcohol consumption, intake frequencies of red meat, intake frequencies of fresh fruit, prevalent diabetes at baseline, leisure-sedentary time and BP-levels.

**Appendix 3. Hazard ratios (95% CI) of CVD across quartiles of total physical activity for all participants and stratified by gender with sensitivity analyses**

| Physical activity | No. of Observations | No. of  Cases | Person years of follow-up | Incidence density (1/1000 person-years) | Model 1  Hazard ratio (95%CI) | *P* value | Model 2  Hazard ratio (95%CI) | *P* value |
| --- | --- | --- | --- | --- | --- | --- | --- | --- |
| All | 20031 | 2332 | 210404 | 11.83 |  |  |  |  |
| Q1 | 4852 | 901 | 50088 | 17.99 | 1.00 |  | 1.00 |  |
| Q2 | 5132 | 629 | 54090 | 11.63 | 0.87 (0.78-0.97) | <0.009* | 0.88 (0.79-0.98) | 0.015* |
| Q3  Q4 | 5021  5026 | 425  377 | 53410  52816 | 7.96  7.14 | 0.73 (0.65-0.82)  0.74 (0.65-0.84) | <0.001*  <0.001* | 0.74 (0.65-0.83)  0.76 (0.66-0.87) | <0.001*  <0.001* |
| Male | 8855 | 1086 | 90788 | 11.96 |  |  |  |  |
| Q1 | 2067 | 427 | 20587 | 20.74 | 1.00 |  | 1.00 |  |
| Q2 | 1718 | 209 | 17716 | 11.80 | 0.83 (0.70-0.98) | 0.025* | 0.82 (0.69-0.98) | 0.025* |
| Q3  Q4 | 2132  2938 | 194  256 | 22086  30399 | 8.78  8.42 | 0.72 (0.60-0.86)  0.73 (0.62-0.87) | <0.001*  <0.001* | 0.71 (0.59-0.85)  0.73 (0.61-0.87) | <0.001*  <0.001* |
| Female | 11176 | 1246 | 119616 | 10.42 |  |  |  |  |
| Q1 | 2785 | 474 | 29501 | 16.07 | 1.00 |  | 1.00 |  |
| Q2 | 3414 | 420 | 36374 | 11.55 | 0.90 (0.79-1.02) | 0.106 | 0.91 (0.80-1.04) | 0.182 |
| Q3  Q4 | 2889  2088 | 231  121 | 31324  22417 | 7.37  5.40 | 0.74 (0.62-0.87)  0.72 (0.59-0.90) | <0.001*  0.003* | 0.76 (0.65-0.90)  0.77 (0.62-0.96) | 0.001*  0.020* |

Model 1 was adjusted for age and gender.

Model 2 was additionally adjusted for smoking status, alcohol consumption, intake frequencies of red meat, intake frequencies of fresh fruit, prevalent diabetes at baseline, leisure-sedentary time and BP-levels.

**Appendix 4. Hazard ratios (95%CI) of CVD across quartiles of total physical activity stratified by age with sensitivity analyses**

|  | No. of Observations | No. of  Cases | Person years of follow-up | Incidence density (1/1000 person-years) | Model 1  Hazard ratio (95%CI) | *P* value | Model 2  Hazard ratio (95%CI) | *P* value |
| --- | --- | --- | --- | --- | --- | --- | --- | --- |
| Age（year） |  |  |  |  |  |  |  |  |
| <50 | 5028 | 178 | 53183 | 3.35 |  |  |  |  |
| Q1 | 462 | 29 | 4975 | 5.83 | 1.00 |  | 1.00 |  |
| Q2 | 1003 | 33 | 10564 | 3.12 | 0.54 (0.33-0.88) | 0.014* | 0.58 (0.34-0.96) | 0.036* |
| Q3  Q4 | 1577  1986 | 45  71 | 16690  20954 | 2.70  3.39 | 0.46 (0.29-0.74)  0.58 (0.38-0.89) | 0.001*  0.013* | 0.50 (0.31-0.82)  0.63 (0.40-1.01) | 0.006*  0.055 |
| 50- | 7337 | 560 | 77893 | 7.19 |  |  |  |  |
| Q1 | 1178 | 107 | 12632 | 8.47 | 1.00 |  | 1.00 |  |
| Q2 | 1969 | 162 | 21006 | 7.71 | 0.92 (0.72-1.17) | 0.485 | 0.96 (0.75-1.23) | 0.738 |
| Q3  Q4 | 2076  2114 | 135  156 | 22103  22152 | 6.11  7.04 | 0.69 (0.53-0.89)  0.74 (0.58-0.96) | 0.004*  0.021* | 0.73 (0.56-0.95)  0.82 (0.63-1.08) | 0.017*  0.153 |
| 60- | 5596 | 984 | 58815 | 16.73 |  |  |  |  |
| Q1 | 2047 | 406 | 21184 | 19.17 | 1.00 |  | 1.00 |  |
| Q2 | 1583 | 261 | 16675 | 15.65 | 0.83 (0.71-0.97) | 0.019* | 0.83 (0.71-0.97) | 0.022* |
| Q3  Q4 | 1134  832 | 190  127 | 12228  8728 | 15.54  14.55 | 0.80 (0.67-0.95)  0.69 (0.57-0.85) | 0.009*  <0.001* | 0.79 (0.67-0.95)  0.70 (0.57-0.87) | 0.010*  0.001* |
| ≥70 | 2070 | 610 | 20513 | 29.74 |  |  |  |  |
| Q1 | 1165 | 359 | 11297 | 31.78 | 1.00 |  | 1.00 |  |
| Q2 | 577 | 173 | 5845 | 29.60 | 0.94 (0.79-1.13) | 0.538 | 0.95 (0.79-1.14) | 0.584 |
| Q3  Q4 | 234  94 | 55  23 | 2389  982 | 23.02  23.42 | 0.70 (0.53-0.93)  0.70 (0.46-1.06) | 0.015*  0.092 | 0.70 (0.52-0.93)  0.71 (0.46-1.08) | 0.013*  0.112 |

Model 1 was adjusted for gender.

Model 2 was additionally adjusted for smoking status, alcohol consumption, intake frequencies of red meat, intake frequencies of fresh fruit, prevalent diabetes at baseline, leisure-sedentary time and BP-levels.

**Appendix 5. Hazard ratios (95%CI) of CVD across quartiles of total physical activity stratified by BP-levels with sensitivity analyses**

|  | No. of Observations | No. of  Cases | Person years of follow-up | Incidence density (1/1000 person-years) | Model 1  Hazard ratio (95%CI) | *P* value | Model 2  Hazard ratio (95%CI) | *P* value |
| --- | --- | --- | --- | --- | --- | --- | --- | --- |
| BP under control | 3113 | 389 | 32272 | 12.05 |  |  |  |  |
| Q1 | 902 | 161 | 9197 | 17.51 | 1.00 |  | 1.00 |  |
| Q2 | 847 | 112 | 8840 | 12.67 | 0.99 (0.77-1.27) | 0.948 | 1.02 (0.79-1.31) | 0.877 |
| Q3  Q4 | 686  678 | 63  53 | 7195  7040 | 8.76  7.53 | 0.83 (0.61-1.13)  0.76 (0.55-1.06) | 0.237  0.103 | 0.85 (0.62-1.16)  0.78 (0.55-1.09) | 0.299  0.148 |
| Stage 1 hypertension | 11442 | 1069 | 120810 | 8.85 |  |  |  |  |
| Q1 | 2506 | 417 | 26031 | 16.02 | 1.00 |  | 1.00 |  |
| Q2 | 2841 | 280 | 30060 | 9.31 | 0.78 (0.67-0.91) | 0.002* | 0.79 (0.67-0.92) | 0.002* |
| Q3  Q4 | 2958  3137 | 181  191 | 31600  33119 | 5.73  5.77 | 0.59 (0.49-0.71)  0.66 (0.55-0.79) | <0.001*  <0.001* | 0.60 (0.50-0.72)  0.68 (0.56-0.82) | <0.001*  <0.001* |
| Stage 2 hypertension | 4009 | 571 | 42098 | 13.56 |  |  |  |  |
| Q1 | 1038 | 204 | 10763 | 18.95 | 1.00 |  | 1.00 |  |
| Q2 | 1041 | 157 | 10954 | 14.33 | 0.99 (0.80-1.23) | 0.948 | 1.00 (0.81-1.23) | 0.973 |
| Q3  Q4 | 1010  920 | 127  83 | 10740  9641 | 11.82  8.61 | 1.00 (0.79-1.26)  0.82 (0.62-1.08) | 0.997  0.164 | 1.02 (0.81-1.30)  0.85 (0.64-1.14) | 0.847  0.280 |
| Stage 3 hypertension | 1467 | 303 | 15224 | 19.90 |  |  |  |  |
| Q1 | 406 | 119 | 4097 | 29.05 | 1.00 |  | 1.00 |  |
| Q2 | 403 | 80 | 4236 | 18.89 | 0.83 (0.62-1.11) | 0.211 | 0.84 (0.63-1.12) | 0.241 |
| Q3 | 367 | 54 | 3875 | 13.94 | 0.66 (0.47-0.93) | 0.016* | 0.69 (0.49-0.98) | 0.036* |
| Q4 | 291 | 50 | 3016 | 16.58 | 0.94 (0.65-1.36) | 0.736 | 0.96 (0.66-1.40) | 0.831 |

Model 1 was adjusted for age and gender.

Model 2 was additionally adjusted for smoking status, alcohol consumption, intake frequencies of red meat, intake frequencies of fresh fruit, prevalent diabetes at baseline and leisure-sedentary time.

**Appendix 6. Basic characteristics of respondent, non-respondent, death population of Respondent and respondents die of CVD at baseline**

| Characteristics | Respondents  (n=20179) | Non-respondents  (n=945) | Death population of Respondents  (n=1554) | Respondents die of CVD  (n=362) |
| --- | --- | --- | --- | --- |
| Age. y | 56.3±9.8 | 62.8±7.8 | 65.0±8.1 | 66.4±7.6 |
| Male, No. (%) | 8941 (44.3) | 461 (48.8) | 858 (55.2) | 189 (52.2) |
| Treated with antihypertension, No. (%) | 8134 (40.3) | 694 (73.4) | 707 (45.5) | 176 (48.6) |
| Diabetes mellitus, No. (%) | 1690 (8.4) | 156 (16.5) | 197 (12.7) | 37 (10.2) |
| SBP, mmHg | 150.9±18.1 | 148.1±20.4 | 155.8±21.8 | 161.7±24.4 |
| DBP, mmHg | 86.3±9.9 | 83.0±10.4 | 84.9±11.8 | 86.2±13.0 |
| BMI, Kg/m^2^ | 25.0±3.3 | 25.0±3.4 | 23.9±3.5 | 23.7±3.4 |
| Resting heart rate, bpm | 80.8±13.1 | 80.7±14.7 | 80.7±13.5 | 79.7±13.1 |
| Leisure-sedentary time, h/d | 3.1±2.2 | 3.6±2.9 | 3.2±2.5 | 3.2±2.5 |
| Current smoker, No. (%) | 5745 (28.5) | 181 (19.2) | 542 (34.9) | 105 (29.0) |
| Current weekly drinker, No. (%) | 3938 (19.5) | 91 (9.6) | 339 (21.8) | 84 (23.2) |
| Education, No. (%) |  |  |  |  |
| ≤6y | 14149 (70.1) | 687 (72.7) | 1295 (83.3) | 311 (85.9) |
| 7-9y | 4270 (21.2) | 151 (16.0) | 173 (11.1) | 34 (9.4) |
| 10-12y | 1364 (6.7) | 76 (8.0) | 62 (4.0) | 12 (3.3) |
| ≥13y | 396 (2.0) | 31 (3.3) | 24 (1.5) | 5 (1.4) |

Mean and standard deviation, unless specified otherwise.
